# Investigating the Speed and Accuracy of Human Movement Corrections to Visual, Somatosensory, and Tactile Perturbations: Evidence for Distinct Sensorimotor Processes

**DOI:** 10.1523/ENEURO.0548-24.2025

**Published:** 2025-04-03

**Authors:** Sadiya Abdulrabba, Jessica Facchini, Gerome Aleandro Manson

**Affiliations:** School of Kinesiology and Health Studies, Queen’s University, Kingston, Ontario K7L 3N6, Canada

**Keywords:** movement corrections, online control, somatosensory target, tactile target, upper limb, visual target

## Abstract

Humans can adapt their movements in response to expected and unexpected perturbations. The speed and accuracy of these movement corrections may depend on the type of sensory information driving the perception of these perturbations. While previous research has indicated that corrections based on somatosensory information, comprised of proprioceptive and tactile inputs, are faster than corrections based on visual information, other studies have found comparable correction speeds in response to visual and tactile inputs. The purpose of this study was to systematically investigate the latencies (how fast) and magnitudes (how large) of movement corrections in response to perturbations of external visual targets, as well as somatosensory (proprioceptive and tactile) and tactile targets on the non-reaching limb. Participants performed reaching movements to a light-emitting diode (i.e., visual target), the felt position of a brush touching the index finger of the non-reaching hand (i.e., a tactile target), and the index finger of their non-reaching hand (somatosensory target). During some trials, the target was displaced 3 cm away or toward the participant either before or after the movement onset. Participants demonstrated faster and larger corrections to somatosensory target perturbations than to visual or tactile target perturbations. However, corrections to visual targets were more accurate than corrections to tactile targets. These findings support the hypothesis that distinct sensorimotor processes may underlie the adjustments made in response to somatosensory information versus those made in response to visual and tactile information.

## Significance Statement

This study focused on systematically comparing the latencies and magnitudes of corrections in response to visual, somatosensory, and tactile cues. We found that corrections to somatosensory cues, consisting of both proprioceptive and tactile information, were faster and larger than corrections to visual and tactile cues, although visual corrections were more accurate (reduced endpoint error) and precise (reduced endpoint variability) than tactile corrections. These findings support the hypothesis that distinct sensorimotor processes underlie movement corrections across different sensory modalities and emphasize the critical role of proprioceptive feedback in facilitating rapid, online adjustments.

## Introduction

Humans continuously and rapidly adjust their movements in everyday activities. For example, when walking a leashed dog, if the dog suddenly pulls away, we instinctively correct our movements, using our free hand to control the leash and prevent the dog from escaping. These rapid corrections are driven by different sensory inputs: visual cues about the dog's movement, tactile feedback from the leash slipping through the hand, and proprioceptive signals from the arm being pulled. The goal of this experiment was to investigate the speed magnitude and accuracy of movement corrections to visual, somatosensory, and tactile cues.

Previous research has found that corrections to changes in somatosensory information occur faster than corrections driven by changes in visual information (Scott et al., 2015; Scott, 2016; Manson et al., 2019). The speed advantage of somatosensory-based corrections has been attributed to the involvement of spinal reflexes and the employment of rapid subcortical and transcortical networks (Pruszynski et al., 2008; Pruszynski et al., 2011; Nashed et al., 2012; Scott et al., 2015; Scott, 2016; Cross et al., 2024). In contrast, corrections based on changes in visual information involve translating retinal information into motor commands (Buneo and Andersen, 2006). These sensorimotor transformations utilize slower visuomotor loops and are also impacted by visual information about the current limb position (Komilis et al., 1993; Reichenbach et al., 2009). For example, Manson et al. (2019) demonstrated that when reaching with an unseen limb, corrections were faster for somatosensory target perturbations compared with visual target perturbations.

Interestingly, faster corrections in response to somatosensory perturbations appear to be driven more by proprioceptive than tactile information. Previous studies have shown that movement corrections in response to tactile perturbations are not significantly faster or larger than corrections in response to visual perturbations (Pruszynski et al., 2016; Abdulrabba et al., 2022). Both Pruszynski et al. (2016) and Abdulrabba et al. (2022) found that when tactile information in one limb indicates a change in the target's position, the time it takes for the other limb to correct its trajectory is not different than the time taken to make corrections based on visual changes in target position. However, both studies also noted that corrections in response to visual target perturbations were more precise (Pruszynski et al., 2016; Abdulrabba et al., 2022). This similarity in detection speed between visual and tactile motion has been attributed to parallel processing mechanisms across the two modalities (Pack and Bensmaia, 2015). Specifically, both visual and tactile systems obtain motion information through spatiotemporal activation patterns on sensory surfaces (retina and skin), and it has been hypothesized that the brain applies similar computational strategies to process motion across these systems (Douglas and Martin, 1991; Pack and Bensmaia, 2015).

While much research has been devoted to comparing visual- and somatosensory-based corrections (Bernier et al., 2007; Scott et al., 2015; Scott, 2016; Manson et al., 2019) or visual- and tactile-based corrections (Pruszynski et al., 2016; Abdulrabba et al., 2022), few studies have explicitly examined the differences between somatosensory- and tactile-based corrections. In the present study, we aimed to systematically investigate the latencies, magnitudes, and accuracy of movement corrections in response to perturbations of external visual targets, as well as somatosensory (proprioceptive and tactile) and tactile-only targets on the non-reaching limb. If the advantages in correction latency and magnitude for targeting a body location are driven by proprioceptive inputs, we would expect shorter correction latencies and larger correction magnitudes in response to somatosensory target perturbations, compared with tactile and visual target perturbations. Conversely, if these advantages are driven by tactile inputs, then the correction latencies and magnitudes for tactile perturbations should not differ from somatosensory perturbations.

## Materials and Methods

### Participants

Fifteen participants (9 women, ages 18–35 years) were recruited. All participants were right-handed as assessed by the Edinburgh Handedness Questionnaire (adapted from Oldfield, 1971), were self-declared neurologically healthy, and had normal or corrected-to-normal vision. Informed consent was obtained prior to the start of the experiment. The experiment took 1.5 h to complete, and the participants were compensated $15 CAD for their time.

### Apparatus

**Figure 1.**
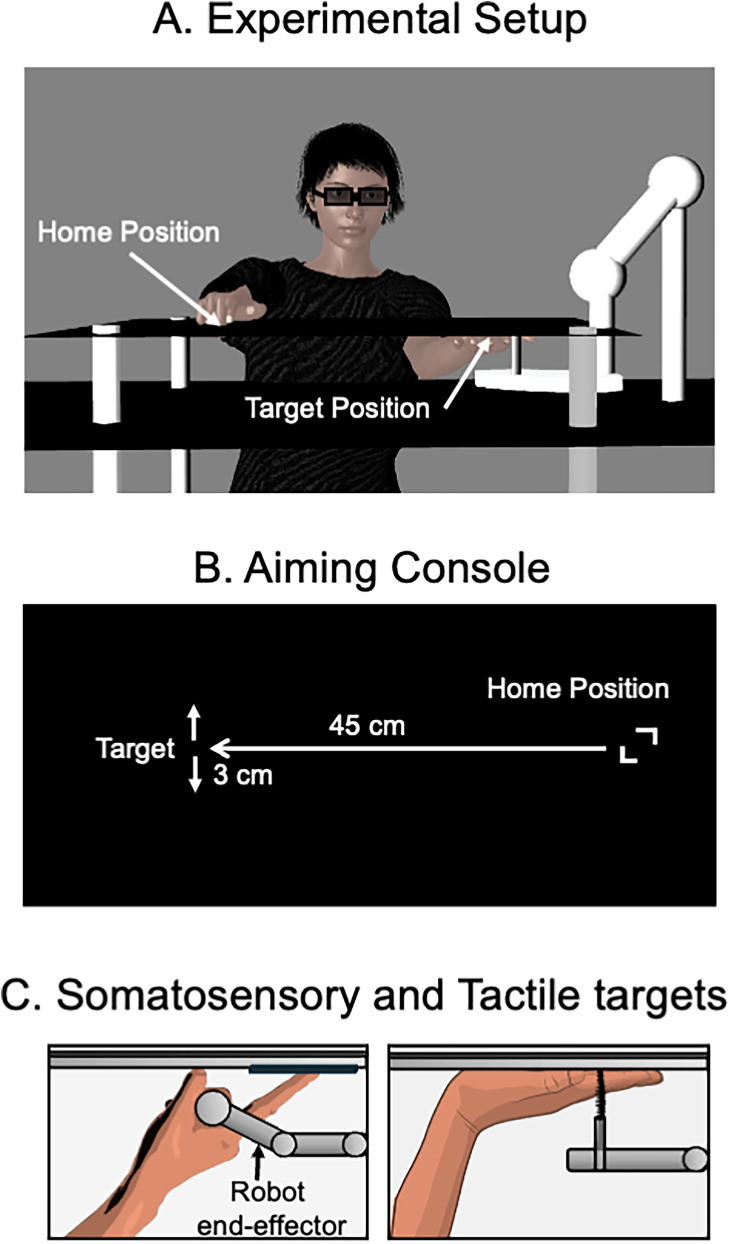
***A***, Experimental setup (not to scale) of the tactile target condition. ***B***, Layout of the aiming console and stimulus positions (not to scale). Participants were seated facing the aiming apparatus in a dark room. Across all target modalities (visual, somatosensory, tactile), a robotic device delivered target perturbations. Participants performed reaching movements from the home position on their right to the target position near their left. ***C***, A representation of the left-hand placement during the somatosensory and tactile conditions. In the visual condition, the left hand was placed beside the aiming console.

The experiment took place in a completely dark room, where participants wore dark glasses (Lincoln Electric KH965) and were seated on an adjustable chair in front of a table (height, 75.5 cm). The dark glasses prevented participants from seeing the dim red light emitted from the infrared emitting diode (IRED) marker on their reaching index finger (see below). Placed on the table was a custom-built aiming surface with a black tinted Plexiglass (60 cm wide by 45 cm long by 0.5 cm thick) mounted 12 cm above a wooden base. A textured home position (2 cm by 2 cm) was located on top of the aiming surface, along with a blue light-emitting diode (LED; ∼6 mm diameter) that served as the fixation (Fig. 1). Placed on the table was a KINOVA Gen3 6 degree-of-freedom robot arm (KINOVA; Gen3, Kinova Robotics).

In the robot's neutral position, a custom end effector attached to the robot arm was positioned below the aiming surface 45 cm left of the home position. In the visual target condition, a green LED (∼6 mm diameter) was attached to the robot's end effector. In the somatosensory target condition, participants were instructed to grasp the robot's end effector with their left hand and place their index finger along a textured target (2 cm by 7 cm) on the underside of the aiming console. The textured target served as a reference for the somatosensory target location (i.e., by providing tactile feedback). In the tactile target condition, participants were instructed to place their left palm on the underside of the aiming console, where a brush (0.5 cm by 2 cm) attached to the robot's end effector was positioned at the center of the medial side of the index finger, at the joint of the proximal and medial phalanxes. During the somatosensory and tactile target conditions, fatigue in the left hand was alleviated by using a support to hold the hand in place below the aiming console. In the visual condition, participants rested their left hand beside the aiming console.

The participant's right index finger (i.e., reaching finger) and the robot arm were affixed with an optical IRED, which was monitored by a Codamotion CX1 (Codamotion) motion tracking system sampling at 400 Hz. A custom MATLAB script (R2021b, MathWorks) was used to send outputs to both the robot effector and collect motion tracking data. A piezoelectric buzzer (SC628, Mallory Sonalert Products) was used to provide the participant with auditory cues for trial start and trial end. The piezoelectric buzzer and the LEDs (i.e., fixation, visual target) were activated using MATLAB (R2021b) and a QUANSER I/O board (DAQ Device Q8-USB).

### Procedure

The experiment included three target modalities (visual, somatosensory, and tactile). The presentation of each target modality was blocked, and the order of blocks was counterbalanced across participants. Reaching movements were made above the aiming surface, and participants were instructed to reach the target as accurately as possible within a movement time bandwidth of 600–800 ms. For all target modalities, the target was presented 45 cm to the left of the home position. The right index finger (i.e., reaching finger) was placed on the home position above the aiming surface.

In each trial, participants performed an overhand reaching movement from the home position to the target position. The signal to begin the movement was a single beep (50 ms). Participants were instructed to keep all fingers on the reaching hand in a fist except for the index finger. The movement time bandwidth was chosen to allow participants sufficient time to make corrections to target perturbations in the trials where the target was perturbed after the movement onset. If the participant's movement time fell outside the movement time bandwidth, they were presented with verbal feedback from the experimenter. At the end of each trial, participants heard two short 50 ms beeps, which signaled them to move back to the home position. Moreover, in trials where the target was perturbed before the movement onset, the movement onset cue delay was fixed and occurred 100 ms after the robot had finished its movement.

For each target modality, there were three types of reaching trials: unperturbed trials, trials where the target was perturbed before movement onset (perturbation before), and trials where the target was perturbed on average 230 ms after the movement onset (perturbation after). In the unperturbed trials, the target remained stationary throughout the trial. In the perturbation trials (perturbation before, perturbation after), the target was perturbed either 3 cm away from or 3 cm toward the participant's body. During the somatosensory target perturbations, the hand was shifted either away from the body (perturbation away) or toward the body (perturbation toward), and participants were instructed to match their right index finger with their left index finger. During the visual target perturbations, the LED was moved either away from or toward the body, and participants were instructed to reach toward the LED. For the tactile target perturbations, a brush was moved along the medial side of the index finger, either distal from the body and toward the fingertip (perturbation away) or closer to the body and toward the palm (perturbation toward). Participants were instructed to reach toward where they perceived the brush alongside their index finger. Two perturbation directions (i.e., away, toward) were used to prevent anticipatory corrections. The movement onset was defined as the time at which the reaching hand was moving above 3 cm/s for 10 ms.

Participants completed a block for each target modality with each block containing 70 trials. The first 10 trials served as familiarization trials, allowing participants to adjust to the target modality (i.e., visual, tactile, somatosensory) and the perturbations (i.e., toward, away) within the modality. The 60 remaining trials were experimental trials, 20 trials with no perturbations (i.e., unperturbed), 20 trials were perturbed away (10 perturbation before, 10 perturbation after), and 20 trials were perturbed toward (10 perturbation before, 10 perturbation after). All unperturbed and perturbed trials were randomized within each block, and the order of target modality conditions was randomized across participants.

Prior to each block, participants completed a calibration block consisting of three trials. During the calibration block, participants were instructed to move to where they perceived the target position at the home position (i.e., unperturbed) and at the perturbed target positions (i.e., 3 cm distal and proximal relative to the target position). In the calibration trials to the perturbed target positions, the target was shifted before the movement onset. The movement endpoints from the calibration block were recorded and used as the reference target position for all endpoint calculations.

### Analyses

#### Data exclusion

Trials were excluded if the participant's endpoint errors in the amplitude axis (i.e., axis of primary movement) or endpoint errors in the direction axis (i.e., axis of target perturbation) exceeded three standard deviations above or below the mean. The means were calculated within each target modality and perturbation type (i.e., away, toward, unperturbed) for each participant. Overall, 6% of all trials were excluded from the analyses.

#### Comparison of unperturbed trials

To examine the effect of target modality on reaching performance, we examined the reaction time, movement time, root mean squared errors (RMSE), and variable errors in the amplitude and direction axes. For each dependent variable, a three-target modality (visual, somatosensory, tactile) one-way ANOVA was conducted.

#### Correction magnitude

Correction magnitude was calculated as the average of the absolute difference between the average endpoint of perturbation trials (before and after) and the average endpoint of the unperturbed trials in the axis of perturbation (i.e., direction axis). Trials were collapsed across away and toward perturbation directions. Correction magnitudes were submitted to a three-target modality (visual, somatosensory, tactile) by two-perturbation-time (before movement onset, following movement onset) repeated-measure ANOVA.

#### RMSE

RMSE was calculated as the average deviation of reaching movement endpoints to the target. The linear distance between the movement endpoints and the target position were calculated; these distances were squared, averaged, and then square-rooted. Two types of RMSE were calculated: RMSE in the axis of perturbation (direction RMSE) and RMSE in the amplitude axis (amplitude RMSE). Trials were collapsed across away and toward perturbation directions. RMSE values were submitted to a three-target modality (visual, somatosensory, tactile) by two-perturbation-time (before movement onset, following movement onset) repeated-measure ANOVA.

#### Variable error

Variable error was calculated as the standard deviation of movement endpoints to each target position. Two types of variable error were computed: variable error in the axis of perturbation (i.e., direction variable error) and variable error in the amplitude axis (i.e., amplitude variable error). Trials were collapsed across away and toward perturbation directions. Variable error values (amplitude, direction) were submitted to three-target modality (visual, somatosensory, tactile) by two-perturbation-time (before movement onset, following movement onset) repeated-measure ANOVA.

#### Correction latency

**Figure 2.**
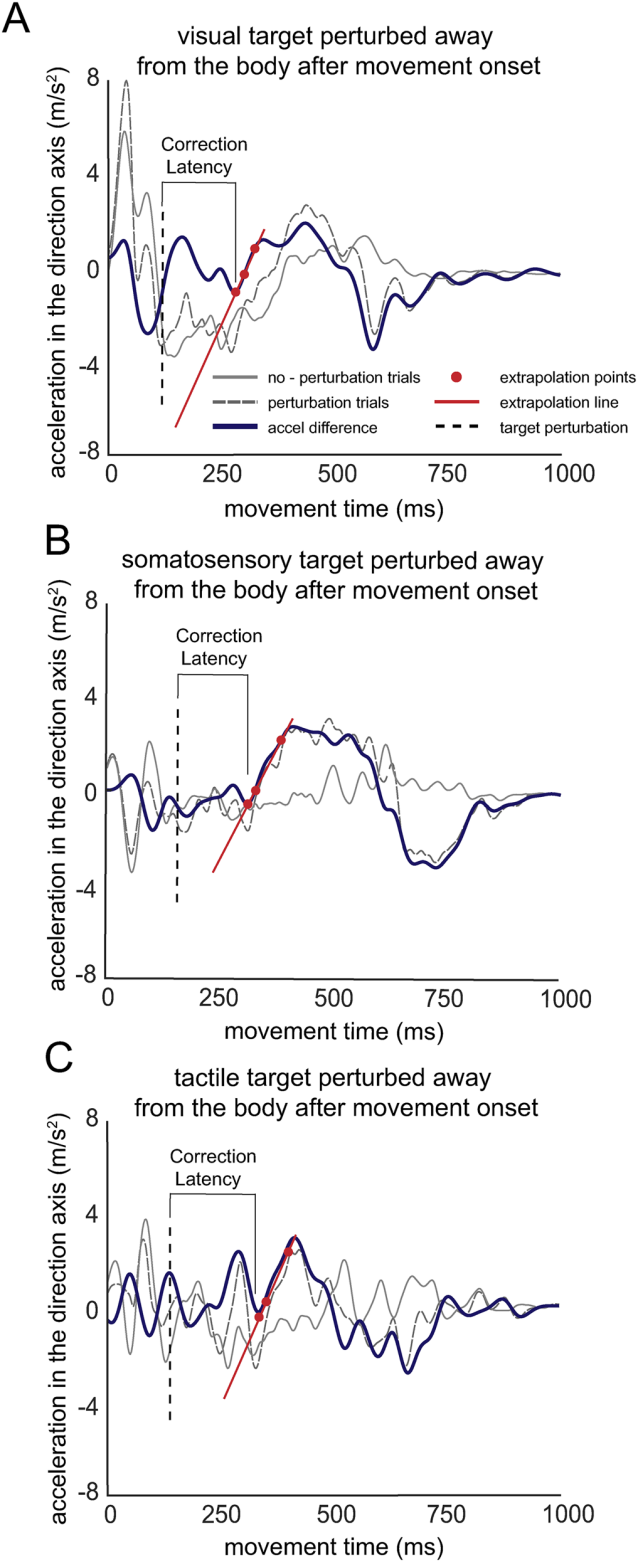
Extrapolation method for determining correction latency. Demonstrated here is the extrapolation data for one participant's movement corrections to a target perturbed away from the body compared with unperturbed trials, across (***A***) visual, (***B***) somatosensory, and (***C***) tactile conditions. Presented on each figure is the average acceleration profile of unperturbed trials (solid gray lines) and perturbation trials (dashed gray lines), along with the difference in acceleration between them (solid blue line). The dashed black line represents the onset of the perturbation. Correction latencies were calculated by drawing an extrapolation line between the 75 and 25% points of the maximum difference in the accel difference profile (extrapolation points) and extending this line to the initial zero crossing. Correction latency is defined as the time from the perturbation onset to this zero crossing.

Correction latency was calculated using acceleration profiles of reaching movements in the axis of perturbation (Wijdenes et al., 2013; Manson et al., 2019; Abdulrabba et al., 2022). For each participant, acceleration data were obtained via double differentiation of the displacement data obtained from sampling the finger IRED. Acceleration data were low-pass filtered using a second-order recursive bidirectional Butterworth filter at 12.5 Hz. Average acceleration was computed between unperturbed trials and perturbation-after trials. These difference profiles were used to compute the correction latencies. To determine correction latency, the maximum acceleration value occurring after perturbation was first identified. Next, a line was drawn between the points on the acceleration profile corresponding to 25 and 75% of the maximum acceleration (Fig. 2). Correction latency was defined as the difference between the time of perturbation and the time instant when this line crossed zero (i.e., *y* value of zero). Correction latencies were submitted to a three-target modality (visual, somatosensory, tactile) one-way ANOVA.[Fig eN-NWR-0548-24F3]

**Figure 3. eN-NWR-0548-24F3:**
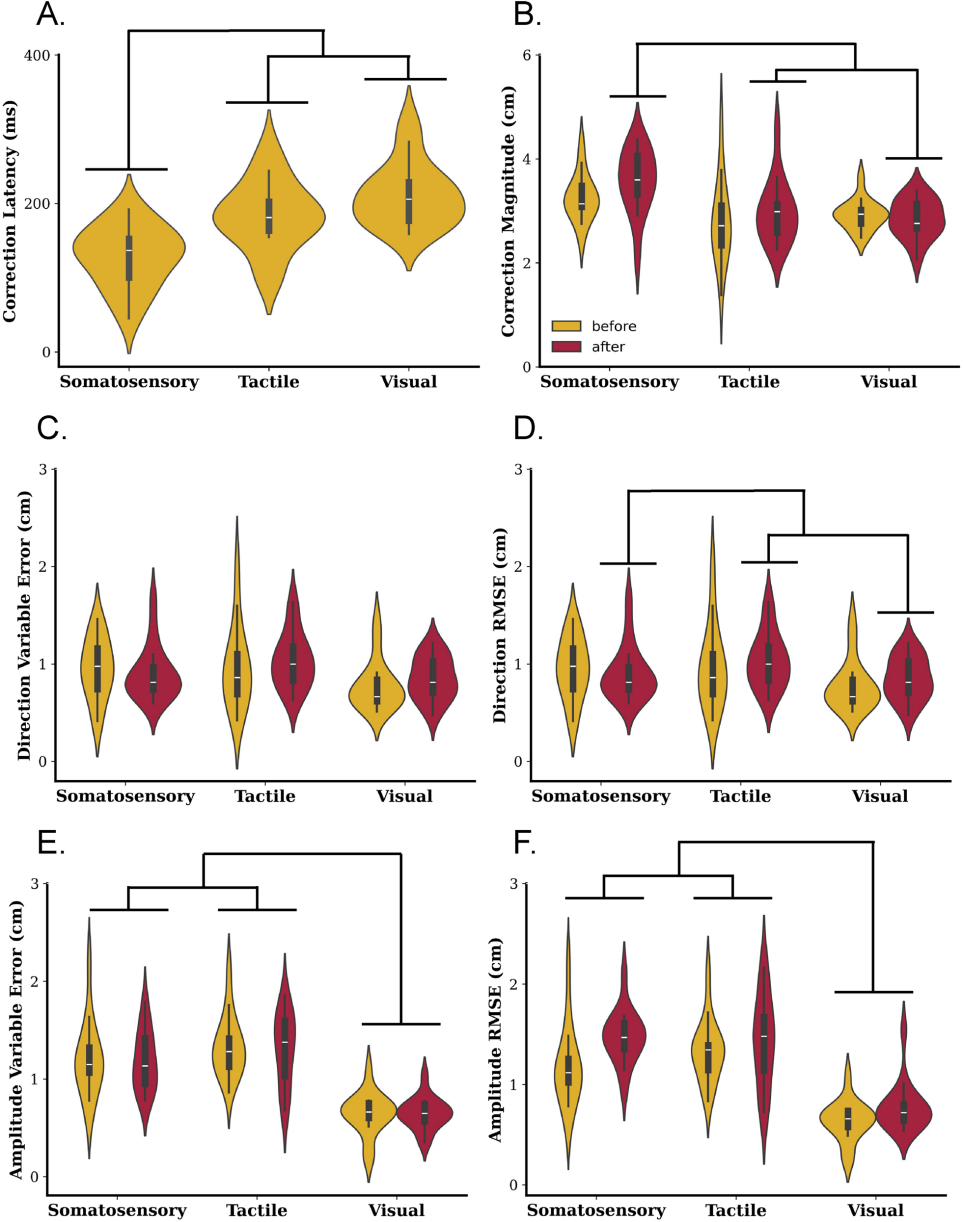
***A***, Correction latency. Participants demonstrated earlier corrections to somatosensory target perturbations compared with tactile and visual target perturbations. ***B***, Correction magnitude. Participants demonstrated larger correction magnitudes to somatosensory target perturbations compared with tactile and visual target perturbations that occurred after the movement onset. ***C***, Direction variable error. Participants demonstrated no differences in direction variable error across sensory conditions. ***D***, Direction RMSE. Participants demonstrated smaller direction RMSE in their movement corrections to visual than somatosensory and tactile target perturbations that occurred after the movement onset. ***E***, Amplitude variable error. Participants demonstrated smaller amplitude variable errors when moving to visual target perturbations compared with somatosensory and tactile target perturbations. ***F***, Amplitude RMSE. Participants demonstrated smaller amplitude RMSE when moving to visual compared with somatosensory and tactile target perturbations. Each plot displays the data distribution with a patch representing the symmetric kernel density estimate. Within each patch is a box plot, with the white line in the box plot representing the data median and the gray box representing the interquartile range (25th to 75th percentiles).

#### Statistical analyses

All statistical analyses were performed using JASP (JASP version 0.18.3, 2024). For all repeated-measure ANOVAs, *α* was set to 0.05. Post hoc comparisons were examined using Bonferroni-corrected *t* tests, whereby the *α* value was adjusted based on the number of comparisons. For clarity and brevity, only the significant main effects and interactions are reported.

## Results

### Comparison of unperturbed trials

Unperturbed trials were analyzed to determine whether the sensory modality of the target had significant effects on the different reaching variables. The analyses revealed significant effects of target modality on reaction time (*F*_(2,28)_ = 47.03; *p *< 0.001; *η*_p_^2 ^= 0.77), amplitude RMSE (*F*_(2,28)_ = 22.77; *p *< 0.001; *η*_p_^2 ^= 0.62), amplitude variable error (*F*_(2,28)_ = 21.19; *p *< 0.001; *η*_p_^2 ^= 0.61), and direction variable error (*F*_(2,28)_ = 3.34; *p *= 0.05; *η*_p_^2 ^= 0.19). Participants took less time to initiate movements to visual targets (M = 387.73 ms; SD = 136.70) compared with somatosensory (M, 615.79 ms; SD, 150.10; *t*_(14)_ = 9.94; *p* < 0.001) and tactile (M, 581.21 ms; SD, 139.52) targets (*t*_(14)_ = 6.95; *p* < 0.001). Smaller amplitude RMSE values were observed when participants reached to visual targets (M, 0.67 cm; SD, 0.18) compared with somatosensory (M, 1.21 cm; SD, 0.30; *t*_(14)_ = 4.54; *p* < 0.001) and tactile (M, 1.41 cm; SD, 0.41) targets (*t*_(14)_ = 6.48; *p* < 0.001). There were also smaller amplitude variable errors when participants reached to visual targets (M, 0.65; SD, 0.18) compared with somatosensory (M, 1.23 cm; SD, 0.32; *t*_(14)_ = 5.66; *p* < 0.001) and tactile (M, 1.37 cm; SD, 0.41) targets (*t*_(14)_ = 6.38; *p* < 0.001). Bonferroni-corrected *t* tests revealed no differences in direction variable error. Moreover, there were no differences in direction RMSE or movement time between target modalities.

### Comparison of perturbed trials

#### Movement time

The analyses of movement time revealed no significant main effects of target modality or perturbation time and no significant interactions (*p*s > 0.05).

#### Reaction time

The analysis for reaction time yielded a significant main effect of target modality (*F*_(2,28)_ = 15.95; *p *< 0.001; *η*_p_^2 ^= 0.53), a significant main effect of perturbation time (*F*_(1,14)_ = 204.02; *p *< 0.001; *η*_p_^2 ^= 0.94), and a significant interaction of target modality and perturbation time (*F*_(2,28)_ = 37.66; *p *< 0.001; *η*_p_^2 ^= 0.73). Participants exhibited shorter reaction times in the visual target perturbation condition (M, 336.28 ms; SD, 142.77) compared with somatosensory (M, 444.19 ms; SD, 135.93; *t*_(14)_ = 5.15; *p* < 0.001) and tactile (M, 439.93 ms; SD, 134.06) target perturbations (*t*_(14)_ = 4.53; *p* < 0.001). There were no differences between the somatosensory and tactile target conditions (*t*_(14)_ = 0.20; *p* = 0.84). Participants exhibited shorter reaction times when the target was perturbed before the movement onset (M, 260.90 ms; SD, 129.35), compared with after the movement onset (M, 552.70 ms; SD, 140.01; *t*_(14)_ = 14.28; *p* < 0.001). Breaking down the interaction, participants exhibited no differences in reaction time across conditions when the target was perturbed before the movement onset (*p *> 0.05). However, there were differences in reaction time across conditions when the target was perturbed after the movement onset. When the target was perturbed after the movement onset, participants exhibited shorter reaction times to visual target perturbations (M, 416.25 ms; SD, 147.17) compared with somatosensory (M, 637.70 ms; SD, 153.87; *t*_(14)_ = 8.29; *p* < 0.001) and tactile (M, 604.15 ms; SD, 158.14) target perturbations (*t*_(14)_ = 5.93; *p* < 0.001). There were no differences in reaction time between somatosensory and tactile targets perturbed after the movement onset (*t*_(14)_ = 1.38; *p* = 0.19).

#### Correction latency

The analysis of correction latency (Fig. 3*A*) yielded a significant main effect of target modality (*F*_(2,28)_ = 19.61; *p* < 0.001; *η*_p_^2^ = 0.58). Participants exhibited earlier corrections to somatosensory target perturbations (M, 128.0 ms; SD, 40.59) compared with visual (M, 211.50; SD = 42.38; *t*_(14)_ = 6.68; *p* < 0.001) and tactile (M, 183.80 ms; SD, 45.74) target perturbations (*t*_(14)_ = 3.89; *p* = 0.002). There were no significant differences between visual and tactile target perturbations (*t*_(14)_ = 1.99; *p* = 0.07).

#### Correction magnitude

The analysis of correction magnitude (Fig. 3*B*) yielded a significant main effect of target modality (*F*_(2,28)_ = 6.69; *p* = 0.004; *η*_p_^2 ^= 0.32), a significant main effect of perturbation time (*F*_(1,14)_ = 4.62; *p *= 0.05; *η*_p_^2 ^= 0.25), and a significant interaction (*F*_(1,14)_ = 3.46; *p *= 0.046; *η*_p_^2 ^= 0.20). Participants exhibited larger corrections to somatosensory target perturbations (M, 3.41 cm; SD, 0.49) compared with visual (M, 2.88 cm; SD, 0.29; *t*_(14)_ = 3.28; *p* = 0.005) and tactile (M, 2.89 cm; SD, 0.66) target perturbations (*t*_(14)_ = 2.72; *p* = 0.017). There were no significant differences between visual and tactile target perturbations (*t*_(14)_ = 0.04; *p* = 0.97). Participants, on average, exhibited smaller corrections when the target was perturbed before the movement onset (M, 2.99 cm; SD, 0.40) compared with after the movement onset (M, 3.14 cm; SD, 0.33; *t*_(14)_ = 2.15; *p* = 0.05). Breaking down the interaction, participants exhibited no differences in correction magnitude when the target was perturbed before the movement onset (*p *> 0.05); however, there were differences in correction magnitude across modalities when the target was perturbed after the movement onset. When the target was perturbed after the movement onset, participants exhibited larger correction magnitudes to somatosensory (M, 3.57 cm; SD, 0.61) compared with visual (M, 2.85 cm; SD, 0.37; *t*_(14)_ = 3.32; *p *= 0.005) and tactile target perturbations (M, 2.99 cm; SD, 0.61; *t*_(14)_ = 2.93; *p *= 0.01).

#### RMSE

The analysis of amplitude RMSE (Fig. 3*F*) yielded a significant main effect of target modality (*F*_(2,28)_ = 29.04; *p* < 0.001; *η*_p_^2 ^= 0.66) and a significant main effect of perturbation time (*F*_(1,14)_ = 7.74; *p *= 0.02; *η*_p_^2 ^= 0.36). Participants exhibited smaller amplitude RMSE to visual targets (M, 0.71 cm; SD, 0.19) than somatosensory (M, 1.33 cm; SD, 0.28; *t*_(14)_ = 7.07; *p* < 0.001) and tactile (M, 1.39 cm; SD, 0.31) targets (*t*_(14)_ = 6.84; *p* < 0.001). There were no significant differences in amplitude RMSE across somatosensory and tactile targets (*t*_(14)_ = 0.55; *p* = 0.59). Participants exhibited larger amplitude RMSE when the target was perturbed after the movement onset (M, 1.23 cm; SD, 0.22) compared with when the target was perturbed before the movement onset (M, 1.05 cm; SD, 0.16; *t*_(14)_ = 2.78; *p* = 0.015).

The analysis of direction RMSE (Fig. 3*D*) yielded a significant main effect of target modality (*F*_(2,28)_ = 4.86; *p* = 0.02; *η*_p_^2 ^= 0.26) and a significant main effect of perturbation time (*F*_(1,14)_ = 18.42; *p *< 0.001; *η*_p_^2 ^= 0.57). Participants exhibited smaller direction RMSE to visual targets (M, 0.83 cm; SD, 0.17) than tactile (M, 1.14 cm; SD, 0.36) target perturbations (*t*_(14)_ = 2.78; *p *= 0.01). There were no differences in direction RMSE between somatosensory (M, 0.99 cm; SD, 0.26) and visual target perturbations and somatosensory and tactile target perturbations (*p* ≥ 0.05). Participants exhibited larger direction RMSE when the target was perturbed after the movement onset (M, 1.09 cm; SD, 0.17) than when the target was perturbed before the movement onset (M, 0.89 cm; SD, 0.20; *t*_(14)_ = 4.29; *p* < 0.001).

Although there was no significant interaction between perturbation time and target modality (*F*_(2,28)_ = 1.90; *p *= 0.17; *η*_p_^2 ^= 0.12), we computed Bonferroni-corrected *t* tests between modalities for direction RMSE for movements where the target was perturbed after the movement onset, as this comparison was relevant to our hypotheses. The results of this comparison showed that corrections in response to visual target perturbations (M, 0.90 cm; SD, 0.16) were significantly more accurate than corrections in response to tactile target perturbations (M, 1.31 cm; SD, 0.38; *t*_(14)_ = 3.47; *p* = 0.004). There were no differences between visual and somatosensory (M, 1.05 cm; SD, 0.35) target perturbations (*t*_(14)_ = 1.58; *p* = 0.14) or somatosensory and tactile target perturbations (*t*_(14)_ = 1.88; *p* = 0.08).

#### Variable error

The analysis for amplitude variable (Fig. 3*E*) error yielded a significant main effect of target modality (*F*_(2,28)_ = 33.40; *p* < 0.001; *η*_p_^2 ^= 0.71). Participants exhibited smaller amplitude variable errors to visual targets (M, 0.64 cm; SD, 0.15), compared with somatosensory (M, 1.19 cm; SD, 0.28; *t*_(14)_ = 6.73; *p* < 0.001) and tactile targets (M, 1.32 cm; SD, 0.25; *t*_(14)_ = 8.03; *p* < 0.001). The analysis for direction variable error yielded no significant main effects of time, condition, or any significant interactions (*p*s > 0.05).

## Discussion

The goal of this experiment was to systematically investigate the latency, magnitude, and accuracy of movement corrections to visual, somatosensory, and tactile target perturbations. Participants performed reaches to visual, somatosensory, and tactile targets that were either stationary or perturbed before or ∼230 ms after the movement onset. We found that participants demonstrated shorter correction latencies and larger correction magnitudes in response to somatosensory target perturbations than tactile and visual target perturbations, which aligns with previous work (Manson et al., 2019; Abdulrabba et al., 2022). Additionally, participants demonstrated greater accuracy and precision when making corrections to visual target perturbations compared with tactile target perturbations. Overall, these results provide evidence that corrections to nonvisual somatosensory perturbations yielded latency and magnitude advantages compared to visual target perturbations; however, these latency and magnitude findings do not extend to nonvisual tactile target perturbations. Moreover, these findings demonstrate that while participants exhibit latency and magnitude advantages when correcting for somatosensory target perturbations, they remain more accurate and precise when correcting for visual target perturbations.

### Participants demonstrated faster and larger corrections to changes in somatosensory target perturbations than visual and tactile target perturbations

Consistent with previous studies, we observed shorter correction latencies in response to somatosensory target perturbations (∼128 ms) than visual target perturbations (∼211 ms). In our study, somatosensory information about changes in the target location was relayed by both proprioceptive and tactile feedback, where changes in arm position were elicited by an external force, and changes in tactile feedback, as the fingertip shifted along a textured surface. The finding that changes in somatosensory targets elicited faster corrections than visual targets aligns with previous experimental observations examining differences in online movement corrections using visual and somatosensory information (Pruszynski et al., 2011; Nashed et al., 2012; Crevecoeur et al., 2016; Scott, 2016; Kasuga et al., 2022).

Rapid corrections in response to changes in proprioceptive targets caused by mechanical perturbations are estimated to occur more quickly than those in response to visual target perturbations. Specifically, stretch reflex-mediated adjustments occur at 25 ms after the perturbation onset, while transcortically mediated adjustments occur at ∼50–60 ms (Pruszynski et al., 2011; Nashed et al., 2012). In contrast, responses to changes in visual information have been estimated to occur relatively later, with initial responses beginning at 90 ms and secondary responses beginning at 130 ms (Nashed et al., 2012; Scott et al., 2015). The between-modality differences in the timing of online corrections have been attributed to the speed of sensorimotor feedback loops. Both neurophysiological and behavioral studies suggest that corrections based on proprioceptive feedback utilize spinal, subcortical, and transcortical networks that operate at faster speeds (i.e., 40–60 ms) than the visuomotor loop (Prevosto et al., 2011; Courjon et al., 2015).

A key difference between previous studies and the current one is that, in previous studies, rapid corrections were triggered using somatosensory information from the reaching limb, while in this study, the somatosensory information used for the corrections came from the non-reaching limb. As a result, participants had to calculate the new target position based on somatosensory input from the non-reaching limb before performing a corrective response with their unseen moving limb. Although the same proprioceptive-based sensorimotor loops could be used, spinal stretch reflex-based corrections likely contributed less to corrective mechanisms than previous studies (Pruszynski et al., 2011; Nashed et al., 2012). The greater reliance on transcortical feedback loops, in combination with the increased complexity of the programmed correction, could explain why the correction latencies in our study are slightly longer than those reported in previous work (Manson et al., 2019). Moreover, unlike previous work, which captured correction latency through changes in muscle activity (Nashed et al., 2012; Pruszynski et al., 2016), our study relied on participant movement acceleration to capture correction latency, which occur much later than changes in muscle activity (Nashed et al., 2012; Wijdenes et al., 2013).

Participants also performed larger corrections in response to somatosensory target perturbations compared with both visual and tactile target perturbations. This finding is consistent with previous research, which has indicated that corrections for somatosensory target perturbations tend to be larger and may overshoot the target position in comparison with corrections to visual target perturbations (Manson et al., 2019). These overcorrections may be due to the reduced spatial sensitivity in the somatosensory target condition in comparison with the visual target condition (see further discussion below).

### Participants were less accurate when correcting to tactile targets after the perturbation than visual targets

In our study, the results indicated that when the targets were perturbed after the start of the movement, corrections made to visual targets were more accurate than those made to tactile targets. One possible explanation may be that the peripheral receptor's capacity to encode spatial information in the retina is superior compared with the tactile receptor's capacity to encode spatial information (Johansson and Vallbo, 1979; Meister and Tessier-Lavigne, 2013). In the tactile condition, participants made reaches toward a tactile cue (i.e., the brush on the finger), which may explain the reduced precision in this condition, compared with the visual condition.

Another potential explanation for the reduced accuracy of tactile corrections could relate to how participants localize these targets. Specifically, participants may localize the target position in visual space prior to the movement onset, as suggested by the longer reaction times observed in the tactile (and somatosensory) target conditions in the unperturbed trials. Although humans generally respond faster to somatosensory stimuli compared with visual stimuli in a simple reaction time tasks (lifting a finger when a stimulus is perceived; Ng and Chan, 2012; Manson et al., 2019; Abdulrabba et al., 2022), reaction times are longer when participants are instructed to move toward a stimulus. Specifically, when moving to tactile or somatosensory targets, reaction times are generally longer than when moving to visual targets (Manson et al., 2019; Abdulrabba et al., 2022). This may be due to additional transformations needed to represent tactile cues in visual space (Brandes and Heed, 2015). In the study by Brandes and Heed (2015), participants performed a movement initiation task and a movement correction task. For movement initiation, participants reached for a visual target after a visual or tactile cue, showing no difference in reaction time across cues. However, in the correction task, corrections to a visual target that appeared after movement onset occurred faster than corrections to a tactile target that appeared after the movement onset, which suggested that additional sensorimotor transformations are required to localize tactile cues in visual space. In this study, movement corrections to somatosensory perturbations may have been faster than movement corrections to visual perturbations due to reflex-mediated adjustments. Yet, this latency advantage did not apply to tactile corrections. Importantly, estimating tactile target locations in visual space may have contributed to the observed reduction in accuracy when localizing and correcting to tactile targets.

### No reliable differences in correction latency between tactile and visual targets

In the present study, there were no differences in the correction latencies to tactile and visual targets. This finding aligns with previous work showing that corrections in response to tactile information are not different than corrections in response to visual information (Pruszynski et al., 2016; Abdulrabba et al., 2022). Research examining the neural processing of tactile and visual motion supports the idea that the time course of corrections to changes in tactile information should not be different than corrections to changes in visual information. In both the visual and tactile systems, motion information is obtained from a spatiotemporal pattern of activation across a sensory sheet (i.e., the retina and the skin; Douglas and Martin, 1991; Pei and Bensmaia, 2014; Pack and Bensmaia, 2015). The brain uses similar computations to calculate motion across the visual and tactile modalities (Douglas and Martin, 1991; Pack and Bensmaia, 2015). Hence, even though the tactile and visual receptors are anatomically different, the brain may use a shared process when interpreting motion information from these very different sensory systems.

There were also no differences in the correction magnitudes to visual and tactile target perturbations, which aligns with previous work (Pruszynski et al., 2016). Here, participants made undercorrections in response to both visual and tactile target perturbations, which was expected, especially with visual-based corrections (Elliott et al., 2010, 2017). Specifically, when making visual-based corrections, individuals tend to undershoot the target location, which has been thought to occur because target overshoots are costlier as they require the limb to travel a greater distance to overcome the inertia from a complete stop at the reversal point (Elliott et al., 2010, 2017).

### Limitations and conclusions

One potential limitation of this study is that we did not include a condition with an exclusively proprioceptive target. Therefore, we cannot rule out the possibility that the changes in correction magnitude and latency were driven by a multisensory combination of tactile and proprioceptive information rather than being attributed solely to proprioceptive inputs. We opted for a somatosensory target because previous literature has shown that tactile cues facilitate the localization of nonvisual targets (Mikula et al., 2018; Mathieu et al., 2022). Future work could address this limitation by examining the contributions of isolated proprioceptive and tactile signals to better understand their independent and combined roles in sensorimotor corrections.

The results of the present study suggest that participants make earlier and larger corrections to somatosensory target perturbations compared with visual and tactile target perturbations. However, participants demonstrate greater accuracy and precision when making corrections to visual target perturbations as compared with tactile target perturbations. These findings support the idea that different sensorimotor processes may underlie the online control of movements to somatosensory compared with visual and tactile targets.

## Synthesis

Reviewing Editor: David Franklin, Technische Universitat Munchen Decisions are customarily a result of the Reviewing Editor and the peer reviewers coming together and discussing their recommendations until a consensus is reached. When revisions are invited, a fact-based synthesis statement explaining their decision and outlining what is needed to prepare a revision will be listed below. The following reviewer(s) agreed to reveal their identity: Tobias Heed.

Your manuscript has been re-reviewed by the two expert reviewers. They both appreciate the revisions that you have made, although we would all have preferred that additional experiments were added to the manuscript to further support the work. Nevertheless, we believe that the manuscript provides some of the first evidence that movement corrections occur to tactile stimuli that move on the body and therefore is an important contribution. We have a few small edits that need to be addressed before the manuscript can be accepted.

Specific comments from the Reviewers

Reviewer 1:

Thank you for revising the manuscript. I find it more clear now. Also the revised figure explaining the setup is helpful.

I have a two remaining points.

1, The reason for why participants wore glasses should be stated in the text, not just in the response letter.

2, there are several instances where a non-significant t-test is claimed to be p=1.00 (an impossible value), however looking up the respective values renders very different values in each case. I suspect this may be due to corrections for multiple testing? If so, it should be stated for how many tests the p-values was corrected. Similarly, lines 311 and 312 state that the p for two different t values is the same (0.23). It is claimed that Bonferroni correction was applied, but I do not understand how the stated values would come about. The true p values for t(14)=1.63 is 0.125; if Bonferroni was applied for 2 tests, then p would be 0.25. It is not obvious to me what other correction may have been applied to arrive at 0.23. The true p-value for t(14)=1.49 is 0.158. Again, it is not clear to me how the 0.23 reported in the manuscript came about. I did not check any other p-values, but I think it would be good to make sure the reported values are indeed correct and to state explicitly how they were arrived at.

To avoid this type of confusion, Bonferroni is often applied in a different way: Rather than adjusting the p-value (which can lead to non-sensical values such as p=1.00 which is impossible for a p-value), the alpha level is adjusted, e.g. by halving p to 0.025 for 2 tests etc.

Reviewer 2:

The authors have adequately addressed my concerns.

FYI- There is a typo in Figure 1: the label 'D' should be 'C'.
